# The prognosis of elderly patients with hepatocellular carcinoma: A multi‐center 19‐year experience in Japan

**DOI:** 10.1002/cam4.4850

**Published:** 2022-05-24

**Authors:** Takeshi Hatanaka, Satoru Kakizaki, Atsushi Hiraoka, Kazuya Kariyama, Kunihiko Tsuji, Toru Ishikawa, Hidenori Toyoda, Satoshi Yasuda, Atsushi Naganuma, Toshifumi Tada, Koichi Takaguchi, Akemi Tsutsui, Ei Itobayashi, Noritomo Shimada, Hiroshi Shibata, Takaaki Tanaka, Takuya Nagano, Michitaka Imai, Shinichiro Nakamura, Kazuhiro Nouso, Hisashi Kosaka, Masaki Kaibori, Takashi Kumada, Real‐life Practice Experts for HCC (RELPEC) Study Group

**Affiliations:** ^1^ Department of Gastroenterology Gunma Saiseikai Maebashi Hospital Maebashi Japan; ^2^ Department of Clinical Research National Hospital Organization Takasaki General Medical Center Takasaki Japan; ^3^ Department of Gastroenterology and Hepatology Gunma University Graduate School of Medicine Maebashi Japan; ^4^ Gastroenterology Center Ehime Prefectural Central Hospital Matsuyama Japan; ^5^ Department of Gastroenterology Okayama City Hospital Okayama Japan; ^6^ Center of Gastroenterology Teine Keijinkai Hospital Sapporo Japan; ^7^ Department of Gastroenterology Saiseikai Niigata Hospital Niigata Japan; ^8^ Department of Gastroenterology and Hepatology Ogaki Municipal Hospital Japan; ^9^ Department of Gastroenterology National Hospital Organization Takasaki General Medical Center Takasaki Japan; ^10^ Department of Internal Medicine Japanese Red Cross Himeji Hospital Himeji Japan; ^11^ Department of Hepatology Kagawa Prefectural Central Hospital Takamatsu Japan; ^12^ Department of Gastroenterology Asahi General Hospital Asahi Japan; ^13^ Division of Gastroenterology and Hepatology Otakanomori Hospital Kashiwa Japan; ^14^ Department of Gastroenterology Tokushima Prefectural Central Hospital Tokushima Japan; ^15^ Department of Surgery Kansai Medical University Hirakata Japan; ^16^ Department of Nursing Gifu Kyoritsu University Japan

**Keywords:** albumin‐bilirubin grade, curative treatment, hepatocellular carcinoma, liver‐related death, personalized treatment

## Abstract

**Aims:**

This retrospective study compared the survival between elderly and non‐elderly patients.

**Methods:**

A total of 5545 treatment‐naive patients with hepatocellular carcinoma (HCC) who visited 7 different hospitals from January 2000 to December 2018 were included. Patients ≥80 years old were defined as elderly patients. We divided the patients into three groups based on the timing of the initial treatment: Early, middle, and late periods defined as 2000 to 2005, 2006 to 2012, and 2013 to 2018, respectively.

**Results:**

There were 132 (8.9%), 405 (17.5%), and 388 (22.2%) elderly patients in the early, middle, and late period, respectively, showing a significant increase over time (*p* < 0.001). In both elderly and non‐elderly patients, the median albumin‐bilirubin score significantly improved over time and the diagnosis of HCC was made slightly earlier over time. The median overall survival (OS) in elderly patients was 52.8, 42.0, and 45.6 months in the early, middle, and late period, respectively, without a significant improvement (*p* = 0.17) whereas the OS in non‐elderly patients was significantly improved (*p* < 0.001). The percentage of elderly patients receiving curative treatments did not significantly increase (*p* = 0.43), while that of non‐elderly patients did (*p* = 0.017). Non‐liver‐related death in elderly patients significantly differed among periods (*p* = 0.023), while liver‐related death did not (*p* = 0.050). Liver‐ and non‐liver‐related death in non‐elderly patients significantly differed among periods (*p* < 0.001, *p* = 0.005).

**Conclusions:**

Survival in elderly patients was not improved despite an improvement in their liver function. Curative treatments should be conducted when appropriate after evaluating each elderly patient.

## 1 INTRODUCTION

Primary liver cancer is the sixth‐most frequent malignant tumor and the third leading cause of cancer death worldwide, with approximately 906,000 new cases and 830,000 deaths annually.^1^ Hepatocellular carcinoma (HCC) accounts for 75%–85% of cases.^1^ According to recent guidelines,^2^, ^3^ treatment of HCC, such as hepatic resection, liver transplantation, radiofrequency ablation (RFA), transarterial chemoembolization (TACE), systemic therapy, or best supportive care (BSC), is determined mainly based on the tumor stage and degree of liver function preservation.

Thanks to advances in medical technology and healthcare, the longevity of the general population has increased in high‐ and upper‐middle‐income countries. For instance, in the United States of America, the number of people ≥65 years old markedly increased from 39.6 million in 2009 to 54.1 million in 2019, with an additional 19.6 years of average life expectancy.^4^ In Europe, the average life expectancy increased from 66.9 years in 1960 to 76.8 years in 2015 for males and from 72.3 years to 82.6 years for females.^5^ In general, elderly patients are considered ‘fragile’ due to their cardiopulmonary function, insufficient renal function, comorbidities, and altered drug metabolism, and they are considered more vulnerable to treatment‐related adverse events than younger patients. Accordingly, older patients are likely to receive suboptimal or undertreatment.

It is well known that cancer incidence increases with age,^6^ and in cases of HCC, aging itself is a risk factor relevant to carcinogenesis.^7^, ^8^ Although many factors are considered to have contributed to the improvement in the survival rate of HCC,^9^ whether or not elderly patients can obtain as good a survival benefit as non‐elderly patients remains uncertain.

The present study compared the survival between elderly and non‐elderly patients based on a 19‐year experience.

## 2 METHODS

### 2.1 Patients

In this multicenter retrospective study, we reviewed the medical records of 5545 patients with HCC who visited seven different hospitals (Okayama City Hospital, Ogaki Municipal Hospital, Ehime Prefectural Central Hospital, Teine Keijinkai Hospital, Saiseikai Niigata Hospital, National Hospital Organization Takasaki General Medical Center, and Gunma Saiseikai Maebashi Hospital) from January 2000 to December 2018. We collected the baseline patient information, including their demographic features, underlying liver diseases, serum biochemistry, tumor extent, and initial treatment. Patients were diagnosed based on the pathological findings or typical radiological features of contrast‐enhanced computed tomography (CT), magnetic resonance imaging (MRI), or ultrasonography (US).^10^ All included patients were treatment‐naïve and had not undergone any previous treatment. In general, treatment decision‐making and implementation were conducted based on the discussions with multidisciplinary teams at each local hospital. The patients who were ≥80 years old were defined as elderly patients, while the remaining patients were defined as non‐elderly patients. We divided the patients into three groups based on the timing of initial treatment: Early, period, and late periods defined as 2000 to 2005, 2006 to 2012, and 2013 to 2018, respectively. The entire study protocol was approved by the Institutional Ethics Committee of Ehime Prefectural Central Hospital (No. 27–34). All procedures were done in accordance with the Declaration of Helsinki. The need for written informed consent was waived because of the retrospective nature of the study.

### 2.2 Definition of underlying liver diseases

The underlying liver diseases in all HCC patients were determined as hepatitis B virus (HBV), hepatitis C virus (HCV), HBV + HCV, alcohol‐related, or others. Cases with seropositivity for hepatitis B surface antigen (HBsAg) and positivity for anti‐HCV antibody were attributed to HBV and HCV infection, respectively. Cases with seropositivity for both HBsAg and anti‐HCV antibodies were classified as HBV + HCV, and cases with seronegativity for both markers and with habitual significant alcohol intake were attributed to alcohol‐related liver disease.

### 2.3 The assessment of the liver function, evaluation of the tumor stage, and definition of liver‐related death

Before the initial treatment, The severity of the liver function was evaluated by the Child‐Pugh classification, albumin‐bilirubin (ALBI) score,^11^ and modified (ALBI) grade.^12^ The ALBI score was calculated using the following formula: ALBI score = (log_10_ bilirubin [μmol/L] × 0.66) + (albumin [g/L] × −0.085).^11^ The mALBI grade was determined by calculating the ALBI score (≤ −2.60: Grade 1, > −2.60 to −2.27: Grade 2a, > −2.27 to −1.39: Grade 2b, > −1.39: grade 3).^12^ We assessed the tumor stage according to the BCLC staging system^3^ and tumor node metastasis stage by the Liver Cancer Study Group of Japan 6th edition.^13^ We defined liver‐related death as that mainly due to HCC, liver failure, and bleeding events and non‐liver‐related death as that due to the other causes of death.

### 2.4 Statistical analyses

The categorical values were described as numbers (percentage) and compared using the χ^2^‐test and Fisher's exact when appropriate. Continuous values were described as the median (interquartile range) and compared using the Mann–Whitney *U* test or Kruskal–Wallis test. The overall survival (OS) was defined as the period between the starting date of initial treatment and the date of death or last visit. We generated the Kaplan–Meier curve and compared values using the log‐rank test. A post‐hoc analysis was conducted using the Bonferroni method if significant differences were observed. The Cox proportional hazard model was used to investigate the factors associated with the OS. Gray's test was used to perform the competing risk survival analysis. The Fine‐Gray model was applied to assess the factors relevant to non‐liver‐related death. Because there is a high correlation between BCLC stage and TNM stage, and between Child‐Pugh classification and ALBI grade, we built two models to avoid multicollinearity. *p* values < 0.05 were considered to indicate statistical significance. All statistical analyses were performed using the EZR software program, ver. 1.53 (Saitama Medical Center, Jichi Medical University, Saitama, Japan), which is a graphical user interface for R (The R Foundation for Statistical Computing, Vienna, Austria).^14^

## 3 RESULTS

### 3.1 Characteristics and survival curve of the overall patients

Of the 5545 patients, 1485 (26.8%), 2315 (41.7%), and 1745 (31.5%) underwent initial treatment in the early, middle, and late period, respectively. The characteristics of overall patients are shown in Table 1. The group age increased significantly over time (*p* < 0.001), and there were 132 (8.9%), 405 (17.5%), and 388 (22.2%) elderly patients in the early, middle, and late periods, respectively, showing a significant increase in the proportion of elderly patients over time (*p* < 0.001). The proportion of male patients was roughly consistent over time. The median body mass index, the percentage of obesity and diabetes mellitus (DM) is increasing (*p* < 0.001, *p* < 0.001, and *p* < 0.001, respectively). The percentage of HCV significantly decreased over time, while the proportion of alcohol‐related and other causes significantly increased over time (*p* < 0.001). Regarding the liver function, the percentage of patients with Child‐Pugh class A significantly increased over time, going from 68.8% in the early period to 70.8% in the middle period to 76.3% in the late period (*p* < 0.001). The median ALBI score also significantly improved over time (*p* < 0.001), resulting in an increase in the proportion of mALBI grade 1 and a decrease in the proportions of mALBI grade 2a, 2b, and 3 (*p* < 0.001). While the difference was quite small, the HCC stage began to be diagnosed significantly earlier, and the serum level of α‐fetoprotein (AFP) decreased significantly over time. Regarding the initial treatments, the proportion receiving curative treatment showed an increasing trend.

**TABLE 1:** Characteristics of overall patients

Factor	Group	Early period (*n* = 1485)	Middle period (*n* = 2315)	Late period (*n* = 1745)	*p* value
Age, median [IQR]		68.0 [61.0, 74.0]	71.0 [63.0, 77.0]	72.0 [65.0, 79.0]	<0.001
Elderly, *n* (%)	≥80 years	132 (8.9)	405 (17.5)	388 (22.2)	<0.001
Gender, *n* (%)	Male	1094 (73.7)	1668 (72.1)	1231 (70.5)	0.143
BMI, [IQR]^a^		22.5 [20.7, 25.0]	23.1 [21.0, 25.6]	23.6 [21.3, 26.0]	<0.001
Obesity, *n* (%)^a^	BMI≥25	225 (25.1)	631 (30.0)	571 (33.2)	<0.001
	BMI≥30	29 (3.2)	94 (4.5)	106 (6.2)	0.002
Diabetes mellitus, *n* (%)^b^		468 (32.0)	794 (34.8)	659 (38.2)	0.001
Underlying liver disease, *n* (%)^c^	HCV	950 (64.0)	1324 (57.4)	808 (46.4)	<0.001
	HBV	254 (17.1)	322 (14.0)	203 (11.7)	
	HBV + HCV	16 (1.1)	11 (0.5)	11 (0.6)	
	Alcohol	100 (6.7)	207 (9.0)	229 (13.2)	
	Others	165 (11.1)	444 (19.2)	490 (28.1)	
	Viral infection	1220 (82.2)	1657 (71.8)	1022 (58.7)	<0.001
Child‐Pugh class, *n* (%)^d^	A	1021 (68.8)	1637 (70.8)	1330 (76.3)	<0.001
	B	383 (25.8)	529 (22.9)	334 (19.2)	
	C	79 (5.3)	146 (6.3)	79 (4.5)	
ALBI score, median [IQR]^e^		−2.28 [−2.62, −1.88]	−2.35 [−2.73, −1.91]	−2.51 [−2.86, −2.00]	<0.001
mALBI grade, *n* (%)^f^	1	411 (27.8)	792 (34.3)	757 (43.5)	<0.001
	2a	331 (22.4)	470 (20.4)	331 (19.0)	
	2b	600 (40.6)	844 (36.6)	526 (30.2)	
	3	136 (9.2)	201 (8.7)	127 (7.3)	
Number of tumors, *n* (%)^g^	Solitary	851 (57.3)	1343 (58.1)	1074 (61.6)	0.023
	Multiple	634 (42.7)	970 (41.9)	669 (38.4)	
Tumor size (cm), median [IQR]^h^		2.6 [1.9, 4.6]	2.7 [1.7, 5.0]	2.7 [1.7, 5.0]	0.47
BCLC stage, *n* (%)^i^	Very early stage	269 (18.1)	428 (18.5)	359 (20.6)	<0.001
	Early stage	727 (49.0)	1028 (44.5)	759 (43.5)	
	Intermediate stage	239 (16.1)	387 (16.8)	289 (16.6)	
	Advanced stage	159 (10.7)	274 (11.9)	240 (13.8)	
	Terminal stage	89 (6.0)	192 (8.3)	96 (5.5)	
TNM stage, *n* (%)	I	327 (22.0)	562 (24.3)	459 (26.3)	0.004
	II	591 (39.8)	908 (39.2)	694 (39.8)	
	III	383 (25.8)	523 (22.6)	378 (21.7)	
	IVa	150 (10.1)	228 (9.8)	156 (8.9)	
	IVb	34 (2.3)	94 (4.1)	58 (3.3)	
AFP, *n* (%)^j^	<200	1117 (75.8)	1803 (78.9)	1393 (81.1)	0.005
	200–400	76 (5.2)	96 (4.2)	57 (3.3)	
	≥400	281 (19.1)	386 (16.9)	267 (15.6)	
DCP, *n* (%)^k^	<200	906 (62.3)	1364 (60.4)	1074 (63.4)	0.101
	200–400	110 (7.6)	167 (7.4)	97 (5.7)	
	≥400	439 (30.2)	726 (32.2)	523 (30.9)	
Initial treatments, *n* (%)	Resection	404 (27.2)	581 (25.1)	517 (29.6)	<0.001
	Ablation with TACE	149 (10.0)	436 (18.8)	398 (22.8)	
	Ablation alone	429 (28.9)	501 (21.6)	284 (16.3)	
	TACE	291 (19.6)	405 (17.5)	277 (15.9)	
	MTA	0 (0.0)	12 (0.5)	33 (1.9)	
	Others	67 (4.5)	88 (3.8)	67 (3.8)	
	BSC	145 (9.8)	292 (12.6)	169 (9.7)	
Curative treatments, *n* (%)	Yes	982 (66.1)	1518 (65.6)	1199 (68.7)	0.094

*Note*: Curative treatments were defined as resection or ablation with or without TACE.

Data were missing for ^a^825, ^b^77 ^c^11, ^d^7, ^e^19, ^f^19, ^g^4, ^h^10, ^i^10, ^j^69, and ^k^139 patients.

Abbreviations: AFP, α‐fetoprotein; ALBI, albumin‐bilirubin; BCLC, Barcelona Clinic Liver Cancer; BMI, body mass index; BSC, best supportive care; DCP, Des‐γ‐carboxy prothrombin; HBV, hepatitis B virus; HCV, hepatitis C virus; mALBI, modified albumin‐bilirubin; TACE, transarterial chemoembolization; TNM, tumor node metastasis stage; MTA, multi‐molecular target agent.

**FIGURE 1:**
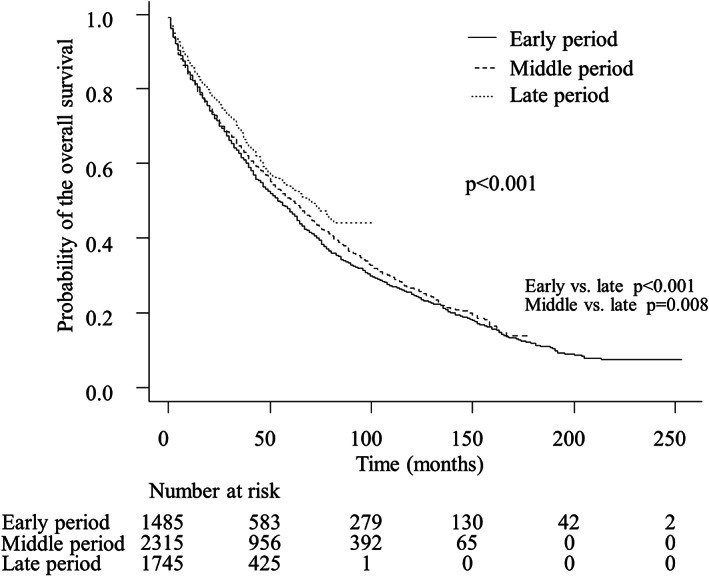
The overall survival according to the three periods in the overall patients

At the time of the analysis, 2957 (53.3%) patients were dead, and the remaining patients were recorded as having been lost to follow‐up or still alive. The median OS in the overall patients in the early, middle, and late period was 54.0 months (95% confidence interval [CI] 49.2–60.0), 61.2 months (95% CI 56.4–66.0), and 70.8 months (95% CI 62.4–79.2), respectively, showing statistical significance (*p* < 0.001). The post‐hoc analysis showed significant differences between the early and late periods (*p* < 0.001) and between the middle and the late period (*p* = 0.008). The 5‐year OS rates in early, middle, and, late period were 47.1% (95% CI 44.2%–49.8%), 50.3% (95% CI 48.0%–52.5%), and 53.9% (95% CI 50.8–56.9%), respectively (Figure 1). In the multivariate analysis, while an elderly age was a significant factor associated with the OS, albeit to a relatively low extent (hazard ratio [HR] of 1.17 in model 1 and 1.11 in model 2), the liver function (Child‐Pugh class and mALBI grade), HCC stage (BCLC stage and TNM stage), and curative treatments greatly contributed to the survival benefit (Table S1).

### 3.2 Characteristics and survival curve of the elderly and non‐elderly patients

**FIGURE 2:**
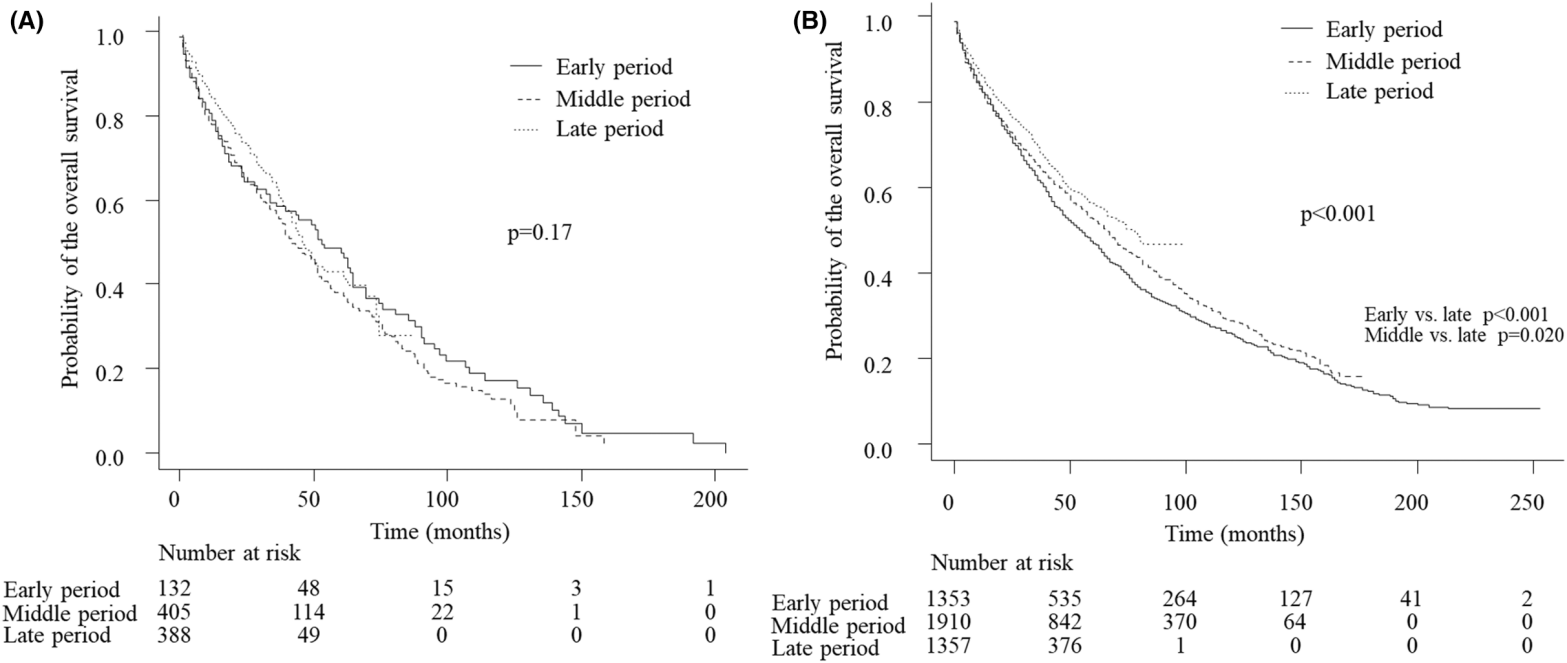
The overall survival according to the three periods in the elderly patients (A) and non‐elderly patients (B). A significant difference was not observed in the elderly patients (*p* = 0.17), but the survival curve significantly differed in the non‐elderly patients (*p* < 0.001)

The characteristics of the elderly patients are described in Table 2. The trends concerning the underlying liver disease, liver function, and BCLC stage were comparable to those in the overall patients. Furthermore, the TNM stage and serum level of AFP were not significantly different between the period, showing similar trends to those of the overall patients as well. The percentage with BSC decreased from 23.5% (*n* = 31) in the early period to 23.0% (*n* = 93) in the middle period to 14.9% (*n* = 58) in the late period. What is particularly striking in this table is that the proportion receiving a curative treatment did not significantly increase over time (*p* = 0.43). The median OS in the early, middle, and late periods was 52.8 months (95% CI 33.6–64.8), 42.0 months (95% CI 37.2–50.4), and 45.6 months (95% CI 42.0–61.2), respectively. The 5‐year OS rates in early, middle, and late periods were 47.2% (95% CI 37.3%–56.5%), 37.6% (32.0%–43.3%), and 42.9% (95% CI 35.3%–50.4%), respectively. Surprisingly, there were no significant differences in OS in the elderly patients among the periods (*p* = 0.17; Figure 2A).

**TABLE 2:** Characteristics of elderly patients

Factor	Group	Early period (*n* = 132)	Middle period (*n* = 405)	Late period (*n* = 388)	*p* value
Age, median [IQR]		82.0 [81.0, 85.0]	82.0 [81.0, 85.0]	83.0 [81.0, 85.0]	0.30
Gender, *n* (%)	Male	82 (62.1)	257 (63.5)	224 (57.7)	0.24
BMI, [IQR]^a^		21.9 [20.0, 24.3]	21.9 [19.9, 24.0]	22.7 [20.3, 25.0]	0.018
Obesity, *n* (%)^a^	BMI≥25	15 (15.6)	64 (18.6)	95 (24.7)	0.048
	BMI≥30	2 (2.1)	9 (2.6)	11 (2.9)	0.91
Diabetes mellitus, *n* (%)^b^		39 (31.0)	131 (33.0)	124 (32.3)	0.91
Underlying liver disease, *n* (%)^c^	HCV	86 (65.2)	238 (58.9)	196 (50.6)	0.003
	HBV	8 (6.1)	16 (4.0)	21 (5.4)	
	HBV + HCV	3 (2.3)	1 (0.2)	1 (0.3)	
	Alcohol	5 (3.8)	21 (5.2)	29 (7.5)	
	Others	30 (22.7)	128 (31.7)	140 (36.2)	
	Viral infection	97 (73.5)	255 (63.1)	218 (56.3)	0.002
Child‐Pugh score, *n* (%)^d^	A	88 (67.2)	292 (72.3)	315 (81.2)	0.006
	B	36 (27.5)	96 (23.8)	59 (15.2)	
	C	7 (5.3)	16 (4.0)	14 (3.6)	
ALBI score, median [IQR]^e^		−2.31 [−2.56, −1.85]	−2.31 [−2.62, −1.91]	−2.47 [−2.74, −2.08]	<0.001
mALBI grade, *n* (%)^f^	1	30 (22.9)	110 (27.3)	155 (40.1)	<0.001
	2a	38 (29.0)	106 (26.3)	84 (21.7)	
	2b	48 (36.6)	157 (39.0)	130 (33.6)	
	3	15 (11.5)	30 (7.4)	18 (4.7)	
Number of tumors, *n* (%)	Solitary	73 (55.3)	266 (65.7)	254 (65.5)	0.075
	Multiple	59 (44.7)	139 (34.3)	134 (34.5)	
Tumor size (cm), median [IQR]^g^		3.0 [2.2, 5.4]	3.1 [2.0, 6.0]	3.2 [1.7, 6.0]	0.47
BCLC stage, *n* (%)^h^	Very early stage	20 (15.3)	55 (13.6)	78 (20.1)	0.037
	Early stage	60 (45.8)	201 (49.8)	155 (39.9)	
	Intermediate stage	30 (22.9)	65 (16.1)	65 (16.8)	
	Advanced stage	14 (10.7)	60 (14.9)	71 (18.3)	
	Terminal stage	7 (5.3)	23 (5.7)	19 (4.9)	
TNM stage, *n* (%)	I	20 (15.2)	85 (21.0)	99 (25.5)	0.23
	II	52 (39.4)	170 (42.0)	152 (39.2)	
	III	38 (28.8)	101 (24.9)	96 (24.7)	
	IVa	18 (13.6)	38 (9.4)	29 (7.5)	
	IVb	4 (3.0)	11 (2.7)	12 (3.1)	
AFP, *n* (%)^i^	<200	97 (74.0)	288 (73.1)	307 (80.4)	0.19
	200–400	6 (4.6)	17 (4.3)	12 (3.1)	
	≥400	28 (21.4)	89 (22.6)	63 (6.5)	
DCP, *n* (%)^j^	<200	65 (52.0)	210 (54.3)	214 (57.2)	0.69
	200–400	10 (8.0)	28 (7.2)	20 (5.3)	
	≥400	50 (40.0)	149 (38.5)	140 (37.4)	
Initial treatments, *n* (%)	Resection	15 (11.4)	59 (14.6)	79 (20.4)	0.002
	Ablation with TACE	12 (9.1)	66 (16.3)	70 (18.0)	
	Ablation alone	40 (30.3)	89 (22.0)	70 (18.0)	
	TACE	32 (24.2)	88 (21.7)	100 (25.8)	
	MTA	0 (0.0)	3 (0.7)	6 (1.5)	
	Others	2 (1.5)	7 (1.7)	5 (1.3)	
	BSC	31 (23.5)	93 (23.0)	58 (14.9)	
Curative treatments, *n* (%)	Yes	67 (50.8)	214 (52.8)	219 (56.4)	0.43

*Note*: Curative treatments were defined as resection or ablation with or without TACE.

Data were missing for ^a^101, ^b^18, ^c^2, ^d^2, ^e^4, ^f^4, ^g^1, ^h^2, ^i^18, and ^j^39 patients.

Abbreviations: AFP, α‐fetoprotein; ALBI, albumin‐bilirubin; BCLC, Barcelona Clinic Liver Cancer; BMI, body mass index; BSC, best supportive care; DCP, Des‐γ‐carboxy prothrombin; HBV, hepatitis B virus; HCV, hepatitis C virus; mALBI, modified albumin‐bilirubin; TACE, transarterial chemoembolization; TNM, tumor node metastasis stage; MTA, multi‐molecular target agent.

The characteristics of non‐elderly patients are described in Table 3. The trends in the etiology of chronic liver disease and the degree of liver function preservation were consistent with those of the overall patients. While the difference was quite small, the HCC stage was diagnosed significantly earlier, and the serum level of AFP decreased significantly over time. The proportion of patients receiving curative treatments increased over time. As expected, the median OS in the early, middle, and late period was 55.2 months (95% CI 48.0%–60.0%), 66.0 months (95% CI 61.2%–70.8%), and 78.0 months (95% CI 66.0–not applicable), respectively, showing statistical significance (*p* < 0.001). A post‐hoc analysis revealed that the extent of significance differed between the early and late periods (*p* < 0.001) and between the middle and the late period (*p* = 0.020). The 5‐year OS rates in the early, middle, and late periods were 47.1% (95% CI 44.1%–50.0%), 52.6% (95% CI 50.1%–55.0%), 56.2% (95% CI 52.8–59.5%), respectively (Figure 2B).

**TABLE 3:** Characteristics of non‐elderly patients

Factor	Group	Early period (*n* = 1353)	Middle period (*n* = 1910)	Late period (*n* = 1357)	*p* value
Age, median [IQR]		67.0 [60.0, 73.0]	68.0 [61.0, 74.0]	69.0 [64.0, 75.0]	<0.001
Gender, *n* (%)	Male	1012 (74.8)	1411 (73.9)	1007 (74.2)	0.84
BMI, [IQR]^a^		22.6 [20.7, 25.1]	23.3 [21.3, 25.8]	23.8 [21.7, 26.2]	<0.001
Obesity, *n* (%)^a^	BMI≥25	210 (26.3)	567 (32.2)	476 (35.6)	<0.001
	BMI≥30	27 (3.4)	85 (4.8)	95 (7.1)	<0.001
Diabetes mellitus, *n* (%)^b^		429 (32.1)	663 (35.2)	535 (39.9)	<0.001
Underlying liver disease, *n* (%)^c^	HCV	864 (63.9)	1086 (57.0)	612 (45.2)	<0.001
	HBV	246 (18.2)	306 (16.1)	182 (13.4)	
	HBV + HCV	13 (1.0)	10 (0.5)	10 (0.7)	
	Alcohol	95 (7.0)	186 (9.8)	200 (14.8)	
	Others	135 (10.0)	316 (16.6)	350 (25.8)	
	Viral infection	1123 (83.0)	1402 (73.6)	804 (59.4)	<0.001
Child‐Pugh score, *n* (%)^d^	A	933 (69.0)	1345 (70.5)	1015 (74.9)	<0.001
	B	347 (25.7)	433 (22.7)	275 (20.3)	
	C	72 (5.3)	130 (6.8)	65 (ss4.8)	
ALBI score, median [IQR]^e^		−2.28 [−2.64, −1.88]	−2.36 [−2.76, −1.90]	−2.52 [−2.87, −1.98]	<0.001
mALBI grade, *n* (%)^f^	1	381 (28.3)	682 (35.8)	602 (44.4)	<0.001
	2a	293 (21.8)	364 (19.1)	247 (18.2)	
	2b	552 (41.0)	687 (36.1)	396 (29.2)	
	3	121 (9.0)	171 (9.0)	109 (8.1)	
Number of tumors, *n* (%)^g^	Solitary	778 (57.5)	1077 (56.4)	820 (60.5)	0.063
	Multiple	575 (42.5)	831 (43.6)	535 (39.5)	
Tumor size (cm), median [IQR]^h^		2.5 [1.8, 4.5]	2.5 [1.7, 4.8]	2.5 [1.6, 4.8]	0.31
BCLC stage, *n* (%)^i^	Very early stage	249 (18.4)	373 (19.6)	281 (20.7)	0.001
	Early stage	667 (49.3)	827 (43.4)	604 (44.6)	
	Intermediate stage	209 (15.5)	322 (16.9)	224 (16.5)	
	Advanced stage	145 (10.7)	214 (11.2)	169 (12.5)	
	Terminal stage	82 (6.1)	169 (8.9)	77 (5.7)	
TNM stage, *n* (%)	I	307 (22.7)	477 (25.0)	360 (26.5)	0.004
	II	539 (39.8)	738 (38.6)	542 (39.9)	
	III	345 (25.5)	422 (22.1)	282 (20.8)	
	IVa	132 (9.8)	190 (9.9)	127 (9.4)	
	IVb	30 (2.2)	83 (4.3)	46 (3.4)	
AFP, *n* (%)^j^	<200	1020 (75.9)	1515 (80.1)	1086 (81.3)	0.006
	200–400	70 (5.2)	79 (4.2)	45 (3.4)	
	≥400	253 (18.8)	297 (15.7)	204 (15.3)	
DCP, *n* (%)^k^	<200	841 (63.2)	1153 (61.7)	860 (65.2)	0.19
	200–400	100 (7.5)	139 (7.4)	77 (5.8)	
	≥400	389 (29.2)	577 (30.9)	383 (29.0)	
Initial treatments, *n* (%)	Resection	389 (28.8)	522 (27.3)	438 (32.3)	<0.001
	Ablation with TACE	137 (10.1)	370 (19.4)	328 (24.2)	
	Ablation alone	389 (28.8)	412 (21.6)	214 (15.8)	
	TACE	259 (19.1)	317 (16.6)	177 (13.0)	

*Note*: Curative treatments were defined as resection or ablation with or without TACE.

Data were missing for ^a^724, ^b^59, ^c^9, ^d^5, ^e^15, ^f^15, ^g^4, ^h^9, ^i^8, ^j^51, and ^k^100 patients.

Abbreviations: AFP, α‐fetoprotein; ALBI, albumin‐bilirubin; BCLC, Barcelona Clinic Liver Cancer; BMI, body mass index; BSC, best supportive care; DCP, Des‐γ‐carboxy prothrombin; HBV, hepatitis B virus; HCV, hepatitis C virus; mALBI, modified albumin‐bilirubin; TACE, transarterial chemoembolization; TNM, tumor node metastasis stage; MTA, multi‐molecular target agent.

### 3.3 Liver‐ and non‐liver‐related death in elderly and non‐elderly patients

Of the 2957 patients who were dead at the time of the analysis, liver‐ and non‐liver‐related death was observed in 1600 (28.9%) and 460 (8.3%), respectively, while the cause of death of 897 (16.2%) patients was not recorded. Among 1600 patients who were judged to liver‐related death, 1190 (21.5%), 370 (6.7%), and 40 (0.7%) patients were dead mainly due to HCC, liver failure, and bleeding events, respectively. The 5‐year cumulative incidence of the liver‐related, non‐liver‐related, and unknown death was 41.2% (95% CI 39.4%–43.0%), 11.5% (95% CI 10.4%–12.7%), and 22.3% (95% CI 20.8%–23.8%), respectively (Figure S1). The cumulative incidence of liver‐related and non‐liver‐related death according to the three periods is shown in Figure S2.

**FIGURE 3:**
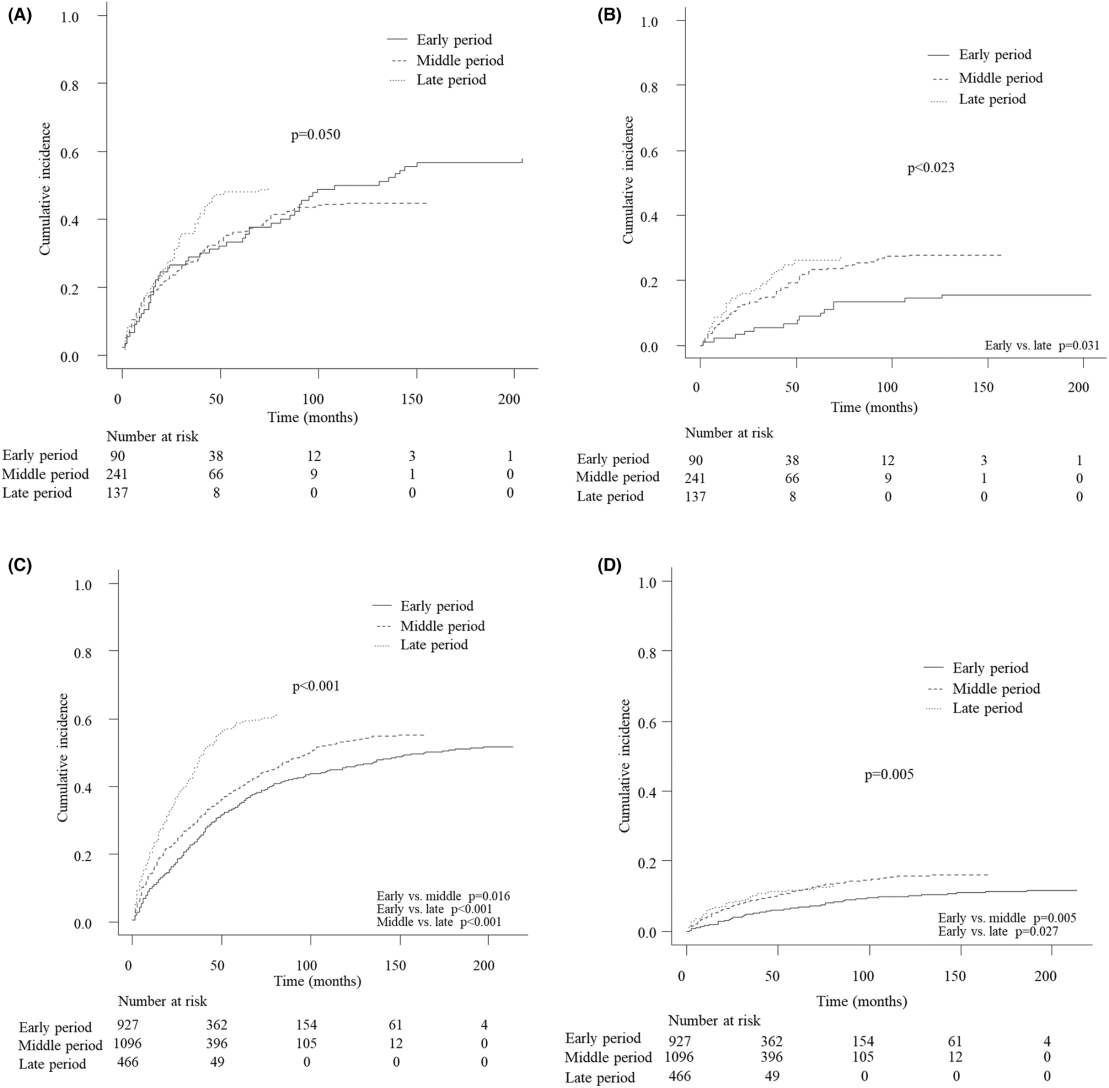
The cumulative incidence of liver‐related death in elderly patients (A), non‐liver‐related death in elderly patients (B), liver‐related death in non‐elderly patients (C), and non‐liver‐related death in non‐elderly patients (D)

In the analysis of the elderly patients, the 5‐year cumulative incidence of liver‐related death was 33.3% (95% CI 23.8%–43.1%) in the early period, 36.1% (95% CI 30.1%–42.1%) in the middle period, and 48.2% (95% CI 39.6%–56.2%) in the late period. Interestingly, a significant difference was not observed (*p* = 0.050; Figure 3A). In contrast, the 5‐year cumulative incidence of the non‐liver‐related death in the early, middle, and late periods was 8.9% (95% CI 4.1–15.9%), 23.2% (95% CI 18.1%–28.7%), and 26.3% (95% CI 19.2%–33.9%, *p* = 0.023), respectively, with a post‐hoc analysis showing a significant difference between the early and late periods (*p* = 0.031; Figure 3B). An elderly age itself was found to be a significant factor associated with non‐liver‐related death, with a relatively large HR (Table S2).

In the analysis of the non‐elderly patients, the 5‐year cumulative incidence of liver‐related death in the early, middle, and late period was 34.9% (95% CI 31.9%–38.0%), 40.0% (37.1%–42.8%), and 58.8% (95% CI 54.2%–63.1%, *p* < 0.001), respectively, with a remarkable absolute increasing. There were significant differences between the early and middle periods (*p* = 0.016), between the early and late periods (*p* < 0.001), and between the middle and late periods (*p* < 0.001) in the post‐hoc analysis (Figure 3C). The 5‐year cumulative incidence of non‐liver‐related death in the early, middle, and late periods was 6.7% (95% CI 5.2%–8.4%), 11.4% (95% CI 9.6%–13.4%), and 11.6% (95% CI 8.9%–14.7%), respectively, resulting in a significant difference (*p* = 0.005) but only a marginal absolute increase. The post‐hoc analysis showed significant differences between the early and middle periods (*p* = 0.005) and between the early and late periods (*p* = 0.027; Figure 3D).

## 4 DISCUSSION

The main findings of the present study were that the median age and percentage of elderly patients were increasing over time, and the liver function was remarkably improved while the diagnosis of HCC was made slightly earlier over time. Surprisingly, we confirmed that the prognosis of the elderly patients did not significantly improve over time, while the survival of the non‐elderly patients was remarkably increased over time. Regarding the non‐elderly patients, the improvement of the liver function and the increase in the curative treatment rate were presumed to largely contribute to the prolongation of the survival curve. However, no survival benefit was observed in the elderly patients, despite an improvement in their liver function and a decrease in the percentage with BSC. One possible reason for this is that the proportion of elderly patients receiving curative treatment, which greatly showed a low HR for the OS in the multivariate analysis, did not significantly increase. Another possible reason is that the non‐liver‐related death among elderly patients differed significantly with the greater absolute difference across the periods, while the liver‐related death did not differed significantly. This finding was also supported by the results of a multivariate analysis showing that an elderly age had a high HR for non‐liver‐related death.

A recent population‐based study^15^ evaluated about 3.9 million patients in seven high‐income countries (Australia, Canada, Denmark, Ireland, New Zealand, Norway, and United Kingdom) with seven primary cancers (esophagus, stomach, colon, rectum, pancreas, lung, and ovary). It showed that the survival benefit was limited in elderly patients, despite advances in the treatment of cancer, while a notable improvement in the OS was observed in younger patients. Although HCC was not assessed, and Japan was not included in that report,^15^ the present study corroborated this previous finding and expanded upon it by showing that the prognosis of elderly patients with HCC did not significantly improve over time.

Regarding curative treatments, surgical resection seems unsuitable for elderly patients because of the increased frequency of postoperative complications. However, many retrospective studies have found that surgical treatment was effective and safe for elderly patients in comparison to younger patients, due to advances in surgical techniques and postoperative management approaches.^16^, ^17^, ^18^ RFA is considered curative treatment for early‐stage HCC and may be feasible for elderly patients owing to its low invasiveness and mild deteriorative effect on the performance status. Two studies noted no significant differences in the OS, local tumor progression, or safety of RFA between elderly and non‐elderly patients.^19^, ^20^ However, another study reported that the OS and local tumor progression were poorer in elderly patients than in non‐elderly patients.^21^ Although data associated with RFA have been conflicting, with careful interpretation required due to the retrospective nature of some analyses, details concerning treatment‐related complications have been consistent, showing that RFA is a safe procedure for elderly patients. Given these findings of studies associated with surgical and RFA treatment, curative treatments should be conducted when appropriate after evaluating each individual case.

Another point of note is that the rate of liver‐related death in elderly patients did not significantly differ among periods, and age itself was strongly associated with non‐liver‐related death, with a high HR in the multivariate analysis. One possible reason for this is that the elderly patients had many comorbidities, leading to an increase in the rate of non‐liver related death. Although the significant extent of the non‐related liver death was presumed to hamper the survival benefit owing to the improvement of the liver function, the present results did not imply that older patients were only suitable for suboptimal or undertreatment. We believe that although precision treatment is always desirable, this is particularly necessary for elderly patients. Curative treatments should include not only proper monitoring to guarantee an adequate treatment intensity but also measures to prevent or minimize the development of adverse events and deterioration of the quality of life. In this connection, the elderly people at the age of 80 years were estimated to be 9.18 years in men and 12.01 years in women according to the life expectancy of the general population.^22^ We also used the life table^23^ to calculate the age‐ and sex‐adjusted mortality rates, showing that the 5‐year survival rate in people ≥80 years old was 94.7% (95% CI 90.1–97.2, data not shown). This calculation indicated that the life expectancy was significantly reduced in HCC patients compared with the general population. From this point of view, we also emphasized the importance of precision treatment regardless of the age.

In the present cohort, the preserved liver function was improved over time. This is mainly because of the development of nucleos (t) ide analog therapy for HBV and direct‐acting antivirals therapies for HCV. Viral infection accounts for the majority of underlying liver disease in the present cohort and these therapies are high efficacy with minimal adverse events, resulting in the improvement of liver function.^24^, ^25^

While the cut‐off for the definition of elderly was varied according to the previous review, ranging from 65 to 85 years, many studies adopted 75 years as the cut‐off value for the definition of elderly.^26^ In the present cohort, the median age in late periods was 72.0 years old and there were many patients ≥75 years (it accounts for 42.2% [*n* = 992]; data was not shown), indicating that many Japanese physician frequently encounter HCC patients ≥75 years in clinical settings. Indeed, the patients aged 75–79 years improved the OS in our analysis (shown in Figure S3). On the other hand, patients ≥80 years accounts for about 20% in late period. Accordingly, we believed that the analysis of patients ≥80 years is important for growing aging society.

While the HBV and HCV infection was still the leading risk factors of carcinogenesis in Asia, the prevalence of HCC due to the metabolic factors is increasing rapidly in Asian countries,^27^ which were consistent with the present results. According to a previous retrospective analysis, the metabolic factors including obesity and DM in non‐viral‐related HCC patients are significantly increasing comparing to the viral‐related HCC patients.^28^ Another study reported that DM is an unfavorable factor associated with OS in early‐stage HCC patients.^29^ The effective surveillance strategies will be required in this special population.

The present study is associated with some limitations. First, this study was conducted in a retrospective manner, and observations in patients who were included in the late period were relatively short compared with those in the early and middle periods. Second, the cause of death was not recorded in about 15% of patients. This might have influenced the present results. Third, we were unable to evaluate the Model for end‐stage liver disease (MELD) score due to the lack of serum creatinine value. A further study to evaluate the utility in MELD score will be conducted.

In conclusion, the percentage of elderly patients is increasing, and the prognosis of elderly patients was not shown to have improved over time. Personalized treatment implemented after evaluating each individual case and considering age‐related comorbidities is desirable, especially in elderly patients.

## AUTHOR CONTRIBUTIONS

Takeshi Hatanaka, Satoru Kakizaki, and Takashi Kumada conceived the study, and participated in its design and coordination. Takeshi Hatanaka, Satoru Kakizaki, Atsushi Hiraoka, Kazuya Kariyama, Kunihiko Tsuji, Toru Ishikawa, Hidenori Toyoda, Satorshi Yasuda, Atsushi Naganuma, Takaaki Tanaka, Michikata Imai, and Kazuhiro Nouso performed data curation. Takeshi Hatanaka performed statistical analyses and interpretation. Toshifumi Tada, Koichi Takaguchi, Akemi Tsutsui, Ei Itobayashi, Noritomo Shimada, Hiroshi Shibata, Takuya Nagano, Shinichiro Nakamura, Hisashi Kosaka, and Masaki Kaibori draft the text. All authors have read and approved the final version of the manuscript.

## FUNDING INFORMATION

There are no funding sources.

## CONFLICT OF INTEREST

Takeshi Hatanaka received lecture fee from Eisai. Atsushi Hiraoka received lecture fee from Eli Lilly, and Chugai. Toshifumi Tada received lecture fee from Abbvie and Eisai. Takashi Kumada received lecture fee from Eisai. None of the other authors have potential conflicts of interest to declare.

## STATEMENT OF ETHICS

The entire study protocol was approved by the Institutional Ethics Committee of Ehime Prefectural Central Hospital (No. 27–34). All procedures were done in accordance with the Declaration of Helsinki. The need for written informed consent was waived because of the retrospective nature of the study.

## Supporting information


Table S1
Table S2

Figure S1

Figure S2

Figure S3

## Data Availability

Due to the nature of this research, patients included in this study could not be contacted regarding whether the findings could be shared publicly, and thus supporting data are not available.
