# Supplementary figures and images for: The prognosis of elderly patients with hepatocellular carcinoma: A multi‐center 19‐year experience in Japan
Source: Cancer Med. 2022 May 24;12(1):345–57. doi: 10.1002/cam4.4850 (PMC9844645; doi:10.1002/cam4.4850)

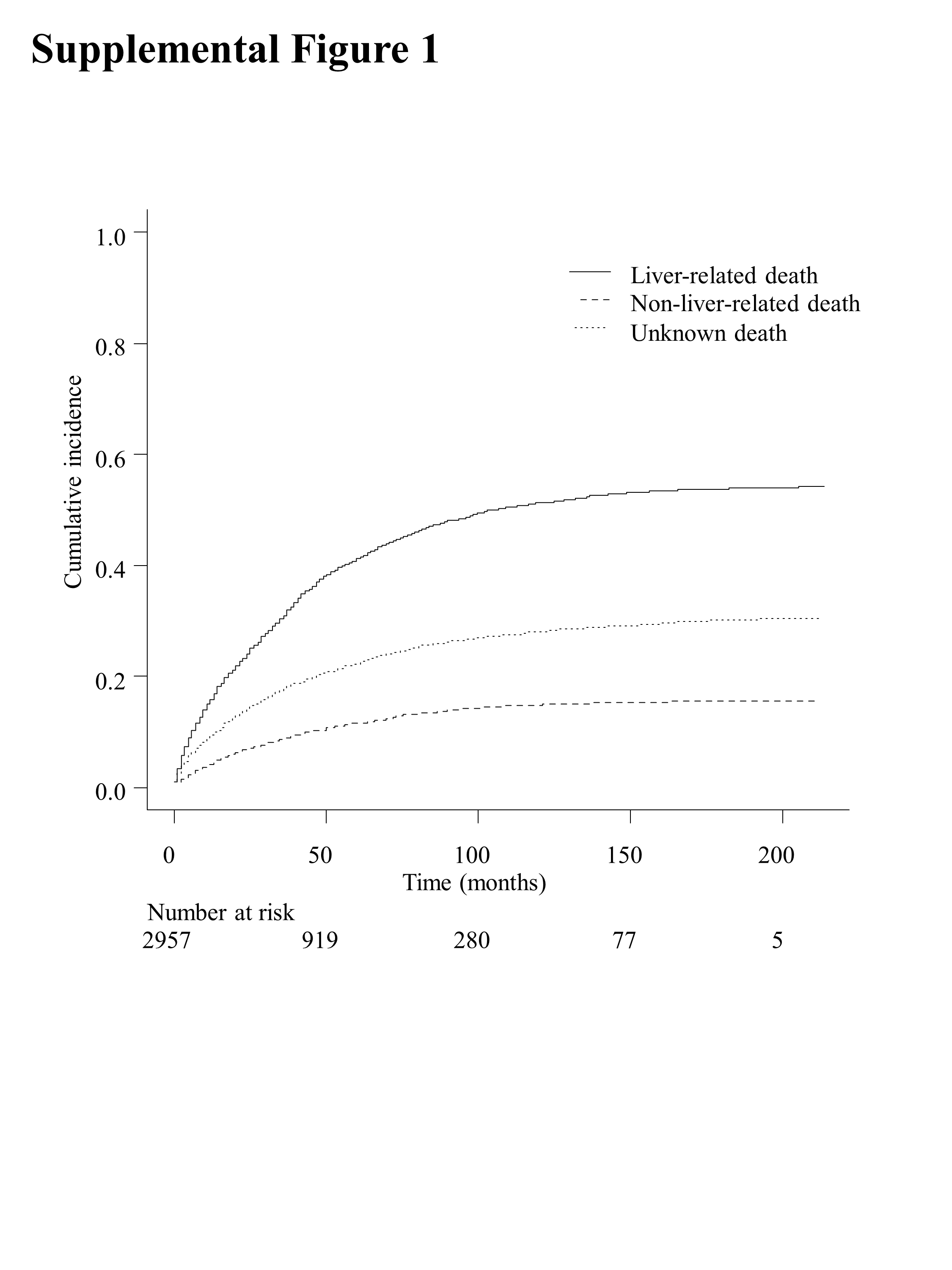

Supplement: Supplementary file 2 — Figure S1 [file CAM4-12-345-s004.TIF]

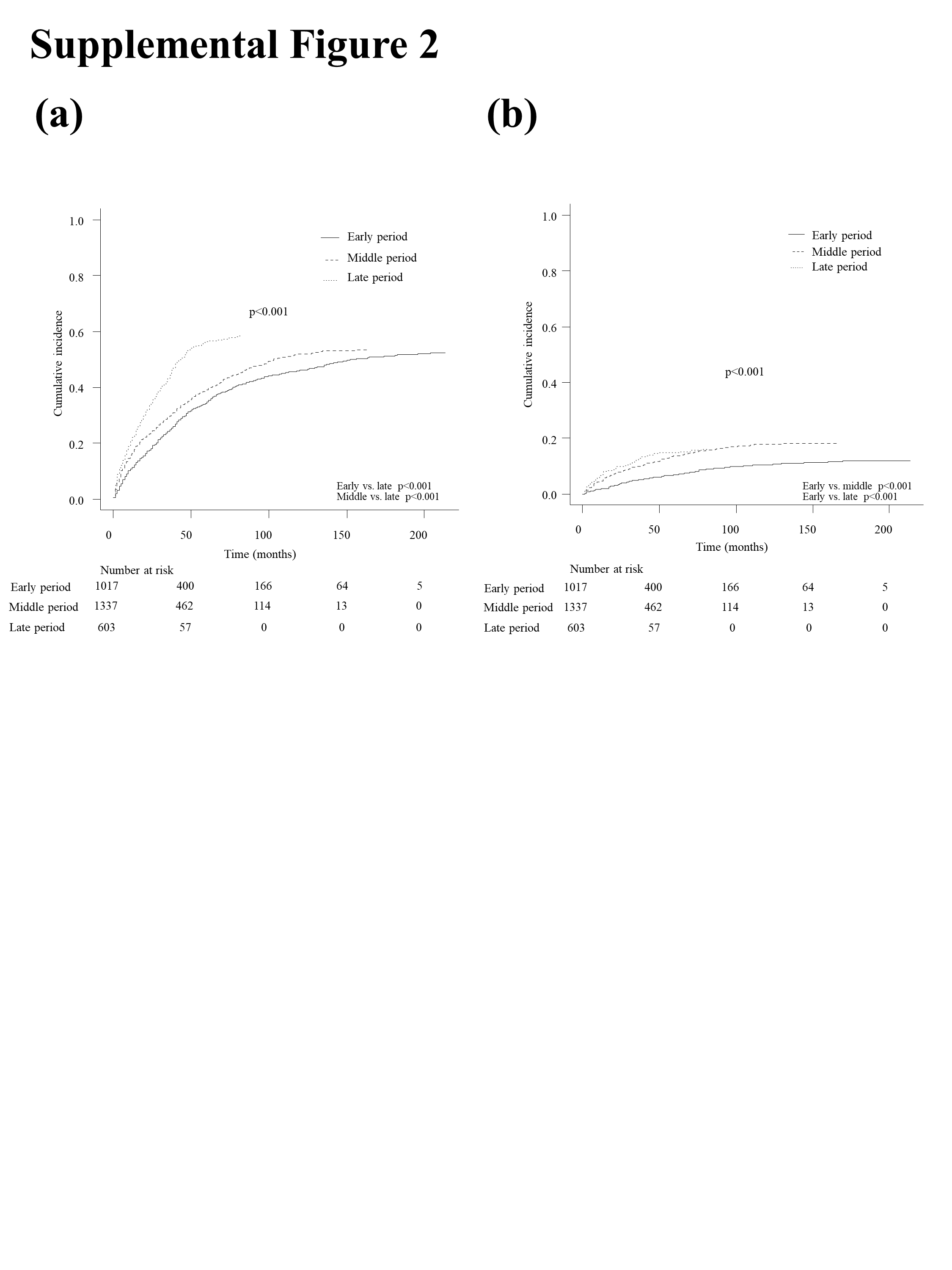

Supplement: Supplementary file 3 — Figure S2 [file CAM4-12-345-s002.TIF]

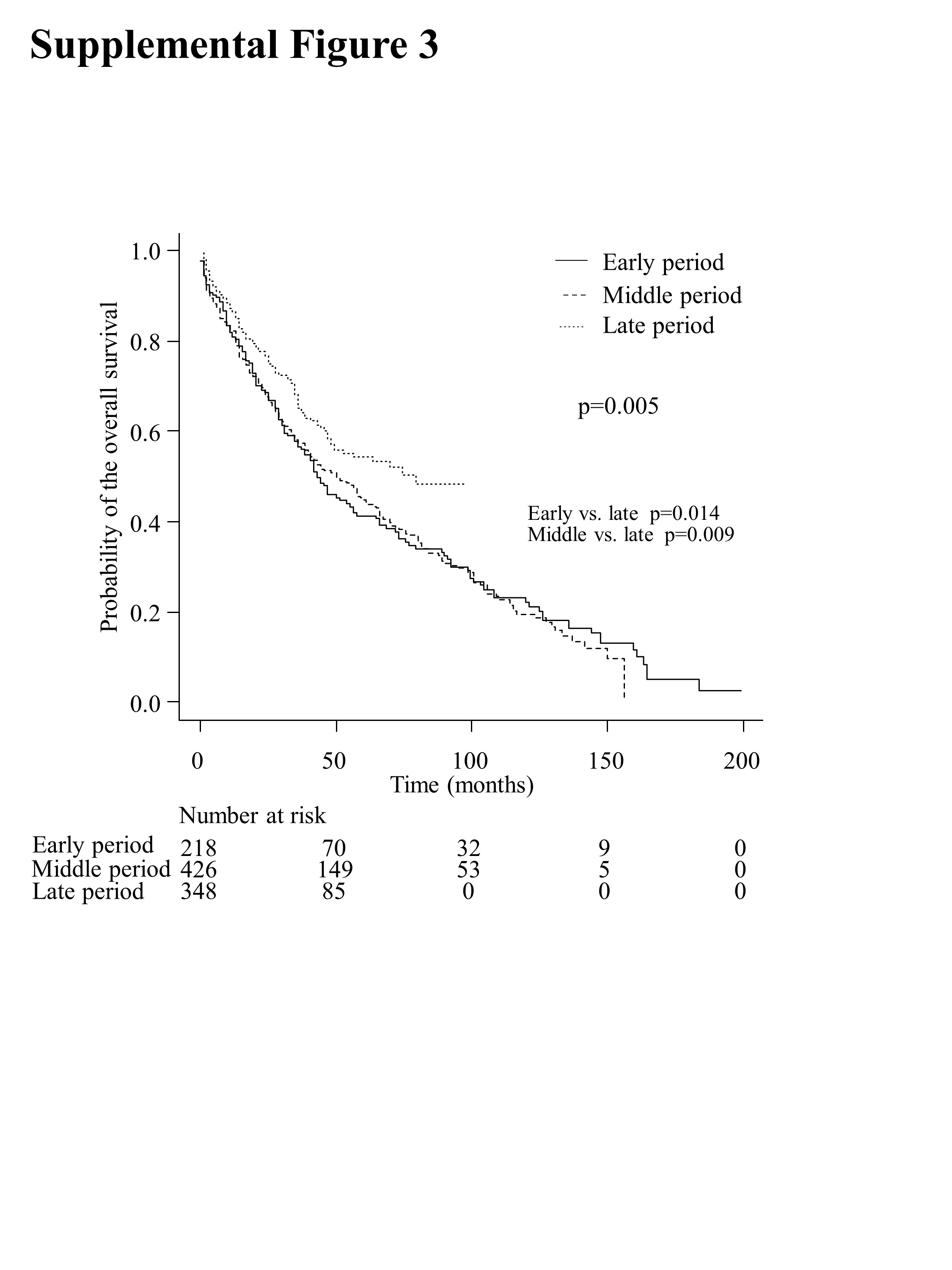

Supplement: Supplementary file 4 — Figure S3 [file CAM4-12-345-s003.TIF]
